# The effect of student-perceived teacher support on math anxiety: chain mediation of teacher–student relationship and math self-efficacy

**DOI:** 10.3389/fpsyg.2024.1333012

**Published:** 2024-04-22

**Authors:** Chao Wang, Qing Xu, Wei-qun Fei

**Affiliations:** ^1^Department of Psychology, School of Teacher Education, Huzhou University, Huzhou, China; ^2^Huzhou Key Laboratory of Brain Science and Child Learning, Huzhou, China; ^3^Department of Psychology, Neuroscience and Behaviour, McMaster University, Hamilton, ON, Canada; ^4^Huzhou Mental Health Education Guidance Center for Primary and Secondary Schools, Huzhou, China

**Keywords:** student-perceived math teacher support, teacher–student relationship, math self-efficacy, math anxiety, elementary school students

## Abstract

**Introduction:**

This study investigates the mechanisms linking students’ perceived teacher support with math anxiety, focusing on the mediating roles of the teacher–student relationship and mathematics self-efficacy.

**Methods:**

The research was conducted with 401 fifth-grade students in China, utilizing scales for Students’ Perceived Teacher Support, Teacher–Student Relationship, Math Self-Efficacy, and Math Anxiety.

**Results:**

Findings revealed that student-perceived math teacher support, teacher–student relationship, and math self-efficacy were all significantly negatively correlated with math anxiety. It was notably found that student-perceived math teacher support influenced math anxiety through the chain mediation of teacher–student relationship and math self-efficacy. Additionally, the effect of students’ perceived emotional support from math teachers on math anxiety, mediated by teacher–student relationship intimacy, was significant only among male students.

**Discussion:**

These results underscore the importance of fostering positive teacher–student interactions and enhancing self-efficacy to reduce math anxiety among primary school students. The gender-specific findings regarding emotional support and relationship intimacy highlight the need for tailored strategies in addressing math anxiety.

## 1 Introduction

Math anxiety is characterized by a state of discomfort when individuals encounter math problems, such as fear, aversion, nervousness, worry, and frustration (Devine et al., 2018; Henschel and Roick, 2018). It stands as one of the prevailing negative academic emotions with cognitive aspects in children and adolescents. Beyond its association with adverse academic outcomes (Wigfield and Meece, 1988; Hembree, 1990; Ho et al., 2000; Ramirez et al., 2013; Ganley and Lubienski, 2016; Ching, 2017), math anxiety significantly influences an individual’s inclination towards STEM (science, technology, engineering, and math)-related career fields (Levy et al., 2021). Studies reveal that math anxiety manifests in children as early as the early primary grades and tends to escalate with age (Devine et al., 2018; Li et al., 2021).

Concerning the causes of math anxiety, extensive global research suggests that it emerges from the interplay of individual factors, such as genetics, neurological function, and cognitive processing, along with environmental factors like teachers, parents, and peers (Luttenberger et al., 2018; Rubinsten et al., 2018). Research on math anxiety in children and adolescents often highlights parents, teachers and peers as primary environmental sources (Gunderson et al., 2012; Casad et al., 2015). Furthermore, teachers play a pivotal role in children’s development, serving as integral participants in the educational process and being the individuals with whom students have the closest contact throughout the day, aside from their parents. Particularly for primary school children, who are also teacher-oriented, the thoughts and behaviors of teachers significantly impact the math anxiety experienced by children’s and adolescents (Tobias, 1978; Kelly et al., 2020). Teacher support, as a positive behavior, plays a crucial role in this context (Wang et al., 2021).

### 1.1 Student-perceived math teacher support

Teacher support is one of the most important forms of social relations that students form and maintain in school (Stevenson et al., 1990; Malecki and Demaray, 2003; Košir and Tement, 2014) and is a crucial source of support for children’s academic achievement (Sakiz et al., 2012). It includes factors such as cognitive support, emotional support, and autonomy support in students’ academic activities. These factors are major influencers of students’ academic emotions and performance (Assor et al., 2005; Richland et al., 2007; Oda et al., 2021). Research indicates that learners’ learning is enhanced in contexts with supportive relationships (Cornelius-White, 2007), and the presence or absence of social support from parents, teachers, and peers can lead individuals to experience various emotional states, including enjoyment, anxiety, and anger (Ahmed et al., 2010). Regrading math anxiety, a negative academic emotion, there is a significant negative correlation with teacher support; that is, students experiencing supportive relationships with their teachers tend to have less math anxiety, whereas those with less supportive relationships tend to have higher math anxiety (Wentzel, 1998; Lapointe et al., 2005; Yıldırım, 2012; Luo et al., 2024).

Measures of teacher support can be categorized based on teacher perspectives and student perspectives. Previous research indicates a divergence in how students and teachers perceive teacher support: students often perceive that high achievers receive more teacher support, while teachers report providing more support to low achievers (Babad, 1990). Stroet et al. (2013) found that students’ perceptions of supportive teaching positively correlate with their overall performance in learning-related tasks. However, in studies focusing on teacher perceptions of support, this correlation is typically weak or nonexistent (Deci, 1975; Entwistle, 1991; Skinner and Belmont, 1993; Opdenakker, 2022). This discrepancy suggests that the impact on students may be more about their perception of teacher behavior than the behavior itself. In essence, the behavior of teachers as perceived by students has a more significant influence on them than the actual behavior of teachers. Therefore, this article will focus on examining teacher support as perceived by students.

### 1.2 Teacher–student relationship

Teachers, playing an important role in educational settings, significantly influence students’ learning through their interactive behaviors. The teacher–student relationship is a key manifestation of this impact. Not only in China, but also in most social environments, fostering internalized socio-emotional relationships is essential for learning, and students particularly value harmonious teacher–student relationships (Fennema and Sherman, 1976; Bao and Lam, 2008; Zhang et al., 2022). The teacher–student relationship encompasses positive interactions between teachers and students and support for students’ learning needs (Collie et al., 2016; Chen et al., 2021).

Other studies have confirmed that students’ perceptions of teacher–student relationships are closely related to their perceived teacher support. Teachers can develop positive relationships by providing such support (Stroet et al., 2013; Zhang et al., 2022). There exists a reciprocal relationship between teacher–student relationships in the classroom and student emotions (Goetz et al., 2021). Positive teacher–student relationships can enhance students’ enjoyment of the course, stimulate positive academic emotions, and reduce math anxiety (Birch and Ladd, 1998; Pinxten et al., 2014; Zee and Roorda, 2018). Conversely, poor teacher–student relationships can lead to a dislike of course-related activities, negative academic emotions, and increased math anxiety. Based on these insights, we propose Hypothesis 1a: Students’ perceived support from math teachers influences math anxiety through the teacher–student relationship. Previous related studies have found significant differences in teacher–student relationships between male and female students. Generally, girls exhibit fewer conflicts with teachers and develop closer student–teacher relationships (Furman and Buhrmester, 1992; Wang and Wang, 2002). Therefore, we propose Hypothesis 1b: Gender has a significant effect on the mediator of student–teacher relationships.

Students’ perceived teacher support and teacher–student relationship are key external factors affecting math anxiety (Luttenberger et al., 2018; Zhou et al., 2020; Li et al., 2021). Math anxiety itself is the result of the interaction between these external factors and internal individual factors, with math self-efficacy being a significant internal factor (Hackett, 1985; Luttenberger et al., 2018; Macmull and Ashkenazi, 2019).

### 1.3 Math self-efficacy

Self-efficacy refers to an individual’s beliefs about their ability to successfully perform a specific task. In the context of math learning, math self-efficacy is an individual’s assessment of their capability to complete mathematical tasks or solve problems successfully. Social cognitive theory posits that perceived support from others enhances an individual’s self-efficacy (Bandura, 1977). In the studies of math self-efficacy, it has been shown that supportive behaviors by math teachers are crucial in influencing students’ math self-efficacy. There is a positive correlation between the support provided by math teachers and students’ math self-efficacy, indicating that higher perceived teacher support is associated with higher math self-efficacy (Wang and Eccles, 2012; Komarraju, 2013; Wu, 2016; Zhang et al., 2019). It has been shown that students with positive attitudes toward learning have more opportunities to engage with their teachers and consequently receive more support (Luo et al., 2024). Additionally, math self-efficacy is associated with positive learning outcomes, suggesting that students with high math self-efficacy are likely to receive more support from their teachers. According to Bandura’s Social Learning Theory, math self-efficacy is a crucial predictor of future math achievement, and as math anxiety is also considered a result of low math self-efficacy, math self-efficacy is a key predictor of future math anxiety (Hackett and Betz, 1981). Analyses based on PISA data (Schulz, 2005; Lee, 2009) have confirmed a significant negative correlation between math self-efficacy and math anxiety, suggesting that higher math self-efficacy correlates with lower math anxiety (Muris, 2002; Macmull and Ashkenazi, 2019; Wang et al., 2023). The investigation into students’ math self-efficacy and anxiety across 41 countries revealed notable differences between Asian and European countries, with Asian countries generally exhibiting lower math self-efficacy and higher levels of math anxiety (Lee, 2009). Math anxiety emerges as early as primary school (Devine et al., 2018; Li et al., 2021). Students in the lower primary grades experience the stress adaptation stage, leading to lower self-efficacy due to academic stress (Zhang et al., 2019). It is not until the sixth grade that students learn to adjust their mindset, resulting in a slight rise in self-efficacy (Shan, 2007). This underscores the importance of exploring whether student-perceived teacher support can alleviate math anxiety by enhancing math self-efficacy. Consequently, we propose Hypothesis 2: Students’ perceived support from math teachers influences math anxiety through math self-efficacy.

Most research on the effects of teacher support on math anxiety has focused on singular social context, with limited exploration into how student-perceived teacher support, an external factor, affects math anxiety through math self-efficacy, an internal factor, affects math anxiety through math self-efficacy, an internal factor. Previous studies have demonstrated that students’ perceived support from teachers contributes to positive teacher–student relationships. Students with positive relationships with their teachers tend to exhibit higher enjoyment and greater self-efficacy in mathematical tasks (Jerome et al., 2009; Sakiz et al., 2012; Zhou et al., 2020; Daliri et al., 2021). Therefore, we propose Hypothesis 3: The teacher–student relationship and math self-efficacy act as chain mediators between students’ perceived teacher support and math anxiety.

**Figure 1:**
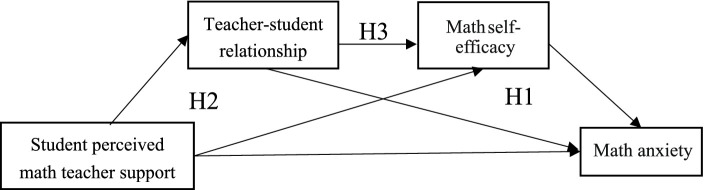
Chain mediation model of students’ perceived math teacher support and math anxiety.

In summary, while prior research has examined the influences on math anxiety, there is a lack of studies exploring how external factors can be transformed into internal ones to alleviate math anxiety. Previous investigations have delved into the relationships among teacher support, teacher–student relationships, mathematics self-efficacy, and math anxiety. However, they have not systematically analyzed the interplay among these four factors. Additionally, most of these studies have focused on secondary school students, even though evidence suggests that math anxiety is also present in primary schools. Therefore, this study aims to address these gaps by exploring the mechanisms through which elementary school students’ perceived math teacher support, teacher–student relationship, and math self-efficacy influence math anxiety, as illustrated in the following model (see Figure 1).

## 2 Methods

### 2.1 Participants

According to Piaget’s stage theory, fifth-grade primary school students are believed to be at a critical stage of transitioning from concrete to formal operations, where their thinking gradually becomes more abstract (Piaget, 1983). This transition is considered crucial for their future math learning and significantly impacts their math anxiety. Accordingly, the participants in this study were 401 fifth-grade students from two public elementary schools in Zhejiang Province in China. This group comprised 189 boys and 212 girls, all aged between 11 and 12 years. These students typically had 5 math classes per week, each lasting 40 min. Due to missing data, one participant was excluded, and an additional 59 participants were excluded because their scale scores deviated by more than two standard deviations from the mean. As a result, 342 valid responses were obtained, resulting in a valid response rate of 85.2%, which included 163 male and 179 female students. Participation in this study was contingent upon obtaining informed consent from the students’ guardians.

### 2.2 Procedure

The scales used in the study underwent a review by the school board before distribution, with the school board assessing the questions to determine their potentially impact on students. Upon receiving approval from the school board, the scales were distributed and collected during students’ study time. All four scales were completed in a single sitting, averaging approximately 15 min for primary school students to complete. The main test was conducted by an individual with a Master’s in Psychology and Education, and instructions were read aloud during the test. The collected data were analyzed using SPSS 26.0 and Process 4.1.

### 2.3 Measures

#### 2.3.1 Student perceived math teacher support

The scale, developed by Chai and Gong (2013) with reference to relevant foreign measurement tools and studies (Skinner and Belmont, 1993; Chen, 2005), was adapted for students’ math learning in China, forming the Students’ Perceived Support for Math Teachers Scale. Proven applicable to elementary school student. The 17-item scale encompasses affective support (e.g., “It is easy for me to communicate with my math teacher”), cognitive support (e.g., “My math teacher asks a series of questions about what she teaches and guides us step by step to find out the answers”), and autonomy support (e.g., “The math teacher gives us enough time to study or think independently”). Employing a five-point Likert scale, scores ranged from 1 to 5, indicating “completely disagree” to “completely agree.” Higher mean scores on each dimension indicate greater support perception. The Cronbach’s alpha coefficient for the overall scale was 0.90, and for affective, cognitive, and autonomy support dimension were 0.75, 0.71, and 0.82, respectively.

#### 2.3.2 Teacher–student relationship

The original Student–Teacher Relationships Scale (STRS) was developed by Pianta (1994) and later revised by Wang and Wang (2002) to include a total of 28 items. Subsequently, Zou et al. (2007) further revised the scale, resulting in a version with 23 items designed to measure students’ perceived teacher–student relationships. This student-rated scale is divided into four dimensions: intimacy, supportiveness, satisfaction, and conflictiveness. The intimacy dimension comprises seven items, such as “The relationship between my math teacher and me is close and warm.” Supportiveness includes five items, for example, “My math teacher helps me in time when I am in trouble.” Satisfaction is assessed with four items, including “The current relationship between my math teacher and me is exactly what I want.” The conflict dimension, consisting of seven items like “My math teacher and I always seem to be struggling with each other,” is reverse-scored. Responses are recorded on a four-point Likert scale ranging from 1 (“not at all”) to 4 (“completely”), where higher scores indicate a more positive perception of the teacher–student relationship. The Cronbach’s alpha reliability coefficients for each dimension ranged from 0.71 to 0.87, demonstrating good internal consistency.

#### 2.3.3 Math self-efficacy

The scale employed in this study was developed by Wang et al. (1999) and is based on relevant dimensions of the Teacher Efficacy Scale originally created by Gibson and Dembo (1984). This scale classifies math self-efficacy into two dimensions: ability and behavior. The first six items assess math ability self-efficacy, with examples like “I have the ability to get good grades in my math homework.” The remaining six items pertain to math behavior self-efficacy, such as “I can easily complete my math homework every day.” Math self-efficacy involves students’ assessment of their capability to complete mathematics tasks, while behavioral self-efficacy in mathematics pertains to students’ assessment of their ability to achieve learning goals in mathematics through personal effort. Responses are recorded on a five-point Likert scale, ranging from 1 (“totally disagree”) to 5 (“totally agree”), where higher scores indicate higher self-efficacy in math. The overall Cronbach’s alpha coefficient for this scale was 0.85. For the dimensions of self-efficacy in mathematical ability and self-efficacy in mathematical behavior, the Cronbach’s alpha coefficients were 0.78 and 0.83, respectively, indicating good internal consistency.

#### 2.3.4 Math anxiety

In this study, we used the Chinese short version of the Mathematics Anxiety Scale (AMAS-C) for elementary school children in grades 1–6. The AMAS-C was revised by Li et al. (2022) based on the AMAS scale developed by Hopko et al. (2003) to align with the cultural context of China. The scale comprises two dimensions: learning math anxiety and math assessment anxiety. The learning math anxiety dimension includes five items, such as “I feel anxious when watching the mathematics teacher answer questions on the board,” and the math assessment anxiety dimension contains four items, like “I feel anxious when thinking about the upcoming mathematics exam.” Responses are gauged on a four-point Likert scale, ranging from 1 (“no anxiety”) to 4 (“great anxiety”), with higher scores indicating higher levels of math anxiety. The Cronbach’s alpha coefficient for the total scale was 0.85. The Cronbach’s alpha coefficients for the dimensions of Math Assessment Anxiety and Learning Math Anxiety were 0.77, and 0.82, respectively.

## 3 Result

### 3.1 Common method bias test

Upon completion of data collection, a common method bias test was conducted using the Harman one-way test. The results revealed 13 factors with eigenvalues greater than 1. However, the first factor explained 26.89 percent of the variance, falling below the critical criterion of 40 percent, indicating the absence of common method bias.

### 3.2 Descriptive statistics and correlation analysis

First, an independent samples *t*-test was conducted to explore the gender differences between the variables (Table 1), revealing significant gender differences (*p* < 0.05) in students’ perceived math teacher support, teacher–student relationship, and math anxiety. Next, correlation analyses were conducted on the main variables, as shown in Table 2. The correlation analyses showed significant positive associations between students’ perceived teacher support and teacher–student relationship, as well as mathematics self-efficacy. Conversely, students’ perceived teacher support exhibited a significant negative correlation with math anxiety. Moreover, teacher–student relationship showed significant positive correlations with mathematics self-efficacy and significant negative correlations with math anxiety. Mathematics self-efficacy, in turn, showed a significant negative relationship with math anxiety. Additionally, as gender is correlated to varying degrees with the four variables under investigation, gender will be examined as a control variable in subsequent analysis.

**Table 1:** Gender differences in variables.

Variable	Gender (M ± SD)		*t*
	Girls (*N* = 179)	Boys (*N* = 163)	
Student Perceived Math Teacher Support	4.54 ± 0.47	4.41 ± 0.53	−2.29^*^
Teacher–student relationship	3.32 ± 0.43	3.17 ± 0.44	−3.14^**^
Mathematics self-efficacy	3.87 ± 0.83	3.90 ± 0.80	0.37
Math anxiety	1.60 ± 0.46	1.71 ± 0.54	2.04^*^

*p < 0.05, **p < 0.01, and ***p < 0.001.

**Table 2:** Descriptive statistics and correlation analysis of variables.

Variable	M ± SD	1	2	3	4	5
1.Gender	–	1				
2.Student Perceived Math Teacher Support	4.48 ± 0.51	−0.12^*^	1			
3.Teacher–student relationship	3.24 ± 0.44	−0.17^**^	0.76^**^	1		
4.Mathematics self-efficacy	3.88 ± 0.81	0.02	0.51^**^	0.52^**^	1	
5.Math anxiety	1.65 ± 0.50	0.11^*^	−0.37^**^	−0.40^**^	−0.66^**^	1

*p < 0.05, **p < 0.01, and ***p < 0.001.

### 3.3 Chain mediation of teacher–student relationship and math self-efficacy

Controlling for gender, mediated effects analyses was conducted using the PROCESS program developed by Hayes. Student-perceived math teacher support served as the predictor variable, math anxiety as the outcome variable, and teacher–student relationship and math self-efficacy as the mediator variables. The results are shown in Table 3. Student-perceived math teacher support significantly and positively predicted teacher–student relationship (*β* = 0.755, *p* < 0.001) and math self-efficacy (*β* = 0.272, *p* < 0.001). Teacher–student relationship significantly and positively predicted math self-efficacy (*β* = 0.326, *p* < 0.001), and math self-efficacy significantly and negatively predicted math anxiety (*β* = −0.631, *p* < 0.001).

**Table 3:** Regression analysis of variables in the model.

		*R* ^2^	*F*	*β*	SE	*t*
Model 1						
TSR	Gender	0.590	243.377	−0.075	0.031	−2.124^*^
	SPMTS			0.755	0.031	21.530^***^
Model 2						
MSE	Gender	0.308	50.224	0.108	0.075	2.354^*^
	SPMTS			0.272	0.113	3.880^***^
	TSR			0.326	0.130	4.614^***^
Model 3						
MA	Gender	0.446	67.832	0.114	0.041	2.744^**^
	SPMTS			0.013	0.063	0.194
	TSR			−0.062	0.074	−0.954
	MSE			−0.631	0.030	−12.949^***^
Model 4						
MA	Gender	0.141	27.803	0.066	0.051	1.292
	SPMT			−0.362	0.050	−7.129^***^

SPMTS, Student Perceived Math Teacher Support; TSR, Teacher–Student Relationship; MSE, Mathematics Self-Efficacy; MA, Math Anxiety. Data reported in the table are standardized coefficients. *p < 0.05, **p < 0.01, ***p < 0.001.

**Figure 2:**
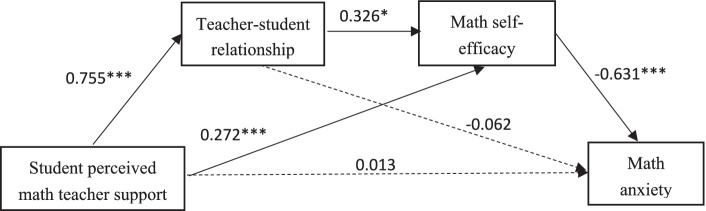
Chain mediation model of students’ perceived math teacher support and math anxiety.

The bias-corrected non-parametric percentile Bootstrap method (5,000 replicated samples) was further used to analyze the mediating effect of the teacher–student relationship and mathematics self-efficacy between students’ perceived mathematics teacher support and math anxiety at a 95% confidence interval. As shown in Table 4, student-perceived mathematics teacher support significantly influenced math anxiety through two paths. Firstly, the effect value of the student-perceived mathematics teacher support → mathematics self-efficacy → math anxiety path was −0.169, with a 95% confidence interval of [−0.262, −0.080]. This interval does not include 0, indicating that the mediation effect is significant, thereby supporting Hypothesis 2. Secondly, the effect value of the student-perceived mathematics teacher support → teacher–student relationship → mathematics self-efficacy → math anxiety path was −0.153, with a 95% confidence interval of [−0.240, −0.074]. This interval also does not include 0, confirming the significance of the mediation effect and establishing Hypothesis 3. These results indicate the chain-mediated effect of student-perceived support from mathematics teachers on math anxiety. In other words, student-perceived support from mathematics teachers not only significantly affects mathematics anxiety through the uni-mediated effect of mathematics self-efficacy, but also indirectly influences math anxiety through the chain-mediated negative effect of the teacher–student relationship, and mathematics self-efficacy (Figure 2). The confidence interval of the direct effect is [−0.112, 0.137]. Since the confidence interval contains zero and the effect value is 0.012, which only accounts for 3.37% of the total effect and is not statistically significant, indicating that teacher–student relationship and math self-efficacy play a fully mediating role between students’ perceived math teacher support and math anxiety.

**Table 4:** Estimated chain-mediated effect sizes of students’ perceived math teacher support on math anxiety.

	Effect	Boot SE	BootLLCI	BootULCI
Total effect	−0.356	0.050	−0.454	−0.258
Direct effect	0.012	0.063	−0.112	0.137
Total indirect effect	−0.368	0.060	−0.487	−0.251
Ind1	−0.046	0.052	−0.152	0.051
Ind2	−0.169	0.047	−0.262	−0.080
Ind3	−0.153	0.042	−0.240	−0.074

Ind1 represents the path Student Perceived Math Teacher Support → Teacher–student relationship → Math anxiety; Ind2 represents the path Student Perceived Math Teacher Support → Mathematics self-efficacy → Math anxiety; Ind3 represents the path Student Perceived Math Teacher Support → Teacher–student relationship →Mathematics self-efficacy → Math anxiety.

In the analysis, it was noted that the effect value of the pathway from student-perceived math teacher’s support → teacher–student relationship → math anxiety was −0.046, with a 95% confidence interval of [−0.152, 0.051].

As this interval includes 0, the mediation effect is not significant, and therefore Hypothesis 1a is not supported. This suggests that student-perceived math teacher support does not significantly influence math anxiety through this path. Consequently, we further examined the chained mediation effects involving three variables along this pathway: students’ perceived mathematics teacher support, the teacher–student relationship, and the sub-dimensions of math anxiety. However, the pathway does not persist when the dimensions are considered independently of gender effects. To investigate the potential influence of gender differences, subsequent analyses were conducted separately for boys and girls based on the dimensional analysis.

The results are shown in Table 5. The effect value of the pathway from boys’ perceived emotional support from mathematics teachers → teacher–student relationship closeness → mathematics anxiety was −0.103, with a 95% confidence interval of [−0.201, −0.015]. The effect value of the pathway from boys’ perceived emotional support from mathematics teachers → mathematics self-efficacy → mathematics anxiety was −0.091, with a 95% confidence interval of [−0.170, −0.015]. The effect value of the pathway from boys’ perceived emotional support from mathematics teachers → teacher–student relationship closeness → math self-efficacy → math anxiety is −0.116, with a 95% confidence interval of [−0.190, −0.056]. None of the aforementioned confidence intervals spans zero, indicating that boys’ perceived emotional support of mathematics teachers within this dimension significantly influences math anxiety through these three pathways. This finding indicates that within the chain of relationships, boys were able to significantly influence math anxiety through the three aforementioned paths, specifically under the dimensions of student-perceived emotional support from mathematics teachers and the closeness of the teacher–student relationship. In contrast, girls did not show a significant influence through these paths, as detailed in Table 6. This indicates that gender does play a role in mediating the pathway of the teacher–student relationship, confirming the validity of Hypothesis 1b.

**Table 5:** Estimated chain-mediated effect sizes for boys’ perceived math teacher support on math anxiety.

	Effect	Boot SE	BootLLCI	BootULCI
Total effect	−0.292	0.053	−0.396	−0.188
Direct effect	0.019	0.059	−0.099	0.136
Total indirect effect	−0.310	0.054	−0.421	−0.212
Ind1	−0.103	0.048	−0.201	−0.015
Ind2	−0.091	0.039	−0.170	−0.015
Ind3	−0.116	0.034	−0.190	−0.056

Ind1 represents the path Student Perceived Math Teacher Support → Teacher–student relationship → Math anxiety; Ind2 represents the path Student Perceived Math Teacher Support → Mathematics self-efficacy → Math anxiety; Ind3 represents the path Student Perceived Math Teacher Support → Teacher–student relationship → Mathematics self-efficacy → Math anxiety.

**Table 6:** Estimated chain-mediated effect sizes for girls’ perceived math teacher support on math anxiety.

	Effect	Boot SE	BootLLCI	BootULCI
Total effect	−0.262	0.048	−0.356	−0.168
Direct effect	0.033	0.063	−0.093	0.158
Total indirect effect	−0.295	0.061	−0.418	−0.180
Ind1	−0.019	0.047	−0.112	0.073
Ind2	−0.199	0.046	−0.295	−0.113
Ind3	−0.077	0.035	−0.151	−0.014

Ind1 represents the path Student Perceived Math Teacher Support → Teacher–student relationship → Math anxiety; Ind2 represents the path Student Perceived Math Teacher Support → Mathematics self-efficacy → Math anxiety; Ind3 represents the path Student Perceived Math Teacher Support → Teacher–student relationship → Mathematics self-efficacy → Math anxiety.

## 4 Discussion

This study investigated the relationship between primary students’ perceived mathematics teacher support, teacher–student relationship, math self-efficacy and math anxiety. It also explored the mechanisms through which teacher–student relationship and mathematics self-efficacy link students’ perceived mathematics teacher support to math anxiety. The results indicated that students’ perceived mathematics teacher support, teacher–student relationship, and mathematics self-efficacy were all significantly negatively correlated with math anxiety. Furthermore, students’ perceived math teacher support was found to significantly and negatively predict math anxiety through the chain-mediated effects of teacher–student relationship and math self-efficacy. Additionally, the study revealed gender differences in the pathway linking students’ perceived mathematics teachers’ emotional support, teacher–student relationship intimacy, and math anxiety. Notably, in the dimensional analysis of students’ perceived math teachers’ support and teacher–student relationship within the chain mediation, significant differences emerged. Girls’ perceived emotional support from math teachers did not significantly and negatively affect their math anxiety through the intimacy of the teacher–student relationship. In contrast, boys’ perceived emotional support from math teachers in the chain significantly and negatively impacted their math anxiety by influencing the closeness of the teacher–student relationship.

### 4.1 Chain mediation of teacher–student relationship and math self-efficacy

This study is the first to explore the roles of teacher–student relationship and math self-efficacy in the context of students’ perceived mathematics teacher support and math anxiety. Consistent with the findings of previous studies (Bandura, 1977; Komarraju, 2013; Stroet et al., 2013), the present study found that teacher support positively predicts teacher–student relationships and mathematics self-efficacy.

Teachers can foster positive and good teacher–student relationships by providing support, and students’ self-efficacy significantly increases in such supportive environments with good teacher–student relationships (Zhou et al., 2020; Daliri et al., 2021). Particularly in primary school, students are drawn to teacher’s recognition and support, fostering a sense of intimacy in the teacher–student relationship. It is important for teachers to encourage students’ enthusiasm and allow them the freedom to make mistakes during the learning process. Employing formative practices, such as providing feedback on students’ strengths and weaknesses in mathematics and guiding them on how to excel, can foster a positive teacher–student relationship and facilitate progress in mathematics. Furthermore, math self-efficacy is also tied to active engagement in learning. Students benefit from participating in practical activities, exploring mathematical knowledge through independent or group activities. Teachers, through emotional and cognitive support, can enhance students’ internal motivation, reduce anxiety, and improve math self-efficacy.

This study also found that teacher–student relationships and mathematics self-efficacy can negatively predict math anxiety, consistent with the number of previous studies (Hackett and Betz, 1981; Birch and Ladd, 1998; Muris, 2002; Macmull and Ashkenazi, 2019). A positive relationship between students and math teachers can reduce students’ negative feelings towards math learning, math classes and activities, thereby reducing math anxiety. Elevated math self-efficacy and increased confidence in their math learning also contribute to a reduction in math anxiety. Notably, the current study found that students’ math self-efficacy plays a more direct role in math anxiety compared to the teacher–student relationship. This suggests that math self-efficacy is particularly crucial in influencing math anxiety. Therefore, mathematics teachers should focus on enhancing students’ math self-efficacy in their teaching practices. In the learning process, teachers should adopt a “student-centered” approach, prioritizing an understanding of each individual student and focusing on their unique needs. At the same time, they should actively acknowledge the significant impact of environmental factors. Teachers ought to cultivate an environment that nurtures student growth by facilitating the transformation of external influences into intrinsic motivation. By establishing close relationships with students, teachers can convert external motivational factors, such as the desire for parental rewards for hard work, into internal motivations. This approach enhances students’ self-efficacy in mathematics and helps reduce math anxiety.

The current study confirms that teacher–student relationship and math self-efficacy play a chain mediating role in students’ perceived teacher support and mathematics anxiety. The significant links between students’ perceived teacher support, teacher–student relationship, mathematics self-efficacy, and math anxiety suggest that students’ math anxiety results from the interaction between environmental and internal factors. Students’ external values can be internalized into intrinsic motivation, mathematics self-efficacy can be enhanced, and math anxiety can be reduced by changing students’ environmental factors. This is consistent with social cognitive theory and self-determination theory (Bandura, 1977; Patrick et al., 2007; Rubinsten et al., 2018). Interestingly, the study found that students’ perceived teacher support does not significantly affect math anxiety solely through the teacher–student relationship. In Luo et al.’s study, it was found that students who perceive high teacher support not only have more positive attitudes towards mathematics, but conversely, students with positive attitudes towards mathematics are also more willing to interact with their teachers, thereby receiving more teacher support (Luo et al., 2024). This suggests that students are not merely passive recipients of environmental influences; influencing and changing students’ environment, including teacher support and the teacher–student relationship, may not suffice to influence students’ math anxiety. It is also crucial to address the interaction between environmental factors and students’ intrinsic characteristics, such as mathematics self-efficacy. Therefore, educators should concentrate on transforming environmental factors influencing students’ math anxiety into intrinsic motivation, ultimately reducing mathematical anxiety and fostering students’ mathematical learning.

An analysis of the dimensions of teacher support and the teacher–student relationship reveals that teacher support primarily enhances the intimacy of the teacher–student relationship through emotional support, thereby reducing students’ math anxiety. Previous studies have suggested that girls often excel in clarity, fluency, and emotionality of verbal expression, maintain more intimate relationships with their teachers, and are more influenced by interpersonal relationships than boys (Furman and Buhrmester, 1992; Wang and Wang, 2002). It might be expected that girls’ perceptions of their math teachers’ emotional support would significantly influence math anxiety through the closeness of the teacher–student relationship. Surprisingly, the current study found that girls’ perceived emotional support from math teachers does not significantly affect math anxiety through teacher–student relationship intimacy. In contrast, boys’ perceived emotional support from math teachers does significantly affect math anxiety through this intimacy. This disparity might arise because girls, while perceiving more interpersonal and emotional support, receive such support from various sources, including peers and parents, not just teachers. Conversely, boys may be more influenced by internal factors such as their own abilities (Levy et al., 2021). Despite these differences, students in general tend to be teacher-oriented, with teachers playing a significant role in their school life. Teachers are pivotal figures in school, and elementary school students naturally seek recognition and support from them (Wentzel, 1998; Malecki and Demaray, 2003; Yıldırım, 2012; Collie et al., 2016). Consequently, the external influences on boys may be more focused on teachers. Contrary to common assumptions that girls are more sensitive and in need of emotional support, with boys often being overlooked in this regard, the results of this study suggest that teachers should provide equal emotional attention and support to both boys and girls.

In summary, students’ perceived support from mathematics teachers had a significant negative predictive effect on math anxiety through the chain of the teacher–student relationship and mathematics self-efficacy. This underscores the need for teachers not only to offer diverse forms of support across various aspects of teaching and learning but also to ensure that these supports go beyond the surface-level assistance. Transforming these external supports into internal motivation (Patrick et al., 2007) and enhancing students’ mathematics self-efficacy can contribute positively to reducing students’ mathematics anxiety.

### 4.2 Research limitations

While this study has contributed to the understanding of the relationship between students’ perceived support from mathematics teachers and math anxiety, it has certain limitations that warrant further investigation. Firstly, as a cross-sectional study, it does not explore whether students’ experiences of teacher–student relationship change with age and how this change may impact math anxiety. A more comprehensive understanding could be achieved through a longitudinal study, investigating age-related variations in teacher–student relationship dynamics and their effects on math anxiety. Secondly, the study primarily focuses on students’ perceived teachers’ support within the school context. Given that social support extends beyond teachers to include significant contributions from parents, it is worth exploring whether parental support can complement teacher support to enhance students’ self-efficacy in mathematics through collaborative efforts between home and school. The potential synergies, mechanisms, and impact of such collaborations on reducing students’ math anxiety merit further investigation and should be considered in further research.

## 5 Conclusion

In conclusion, a significant correlation emerged among students’ perceived mathematics teacher support, teacher–student relationship, mathematics self-efficacy and math anxiety. The study revealed that teacher–student relationship and mathematics self-efficacy played integral roles as chain mediators. Specifically, students’ perceived mathematics teacher support demonstrated a significant negative prediction on math anxiety through the chain mediating effects of teacher–student relationship and mathematics self-efficacy. Further dimensional analyses, considering gender differences, showed that male students’ perceived emotional support from mathematics teachers significantly influenced math anxiety through the closeness of the teacher–student relationship, whereas the impact was non-significant in the case of female students.

## Data availability statement

The raw data supporting the conclusions of this article will be made available by the authors, without undue reservation.

## Ethics statement

The studies involving humans were approved by School of Teacher Education, Huzhou University. The studies were conducted in accordance with the local legislation and institutional requirements. Written informed consent for participation in this study was provided by the participants’ legal guardians/next of kin.

## Author contributions

CW: Conceptualization, Formal analysis, Investigation, Methodology, Supervision, Validation, Visualization, Writing – review & editing. W-qF: Conceptualization, Data curation, Investigation, Methodology, Writing – review & editing. QX: Formal analysis, Investigation, Validation, Visualization, Writing – original draft.
